# Prospective study of ^11^C–methionine PET for distinguishing between recurrent brain metastases and radiation necrosis: limitations of diagnostic accuracy and long-term results of salvage treatment

**DOI:** 10.1186/s12885-017-3702-x

**Published:** 2017-11-06

**Authors:** Shoji Yomo, Kazuhiro Oguchi

**Affiliations:** 1 Division of Radiation Oncology, Aizawa Comprehensive Cancer Center, Aizawa Hospital, 2-5-1, Honjo, Matsumoto-city, Nagano-prefecturem, Japan; 2 Positron Imaging Center, Aizawa Hospital, 2-5-1, Honjo, Matsumoto-city, Nagano-prefecturem, Japan

**Keywords:** ^11^C–methionine, Positron emission tomography, Brain metastases, Radiation necrosis, Local recurrence

## Abstract

**Background:**

On conventional diagnostic imaging, the features of radiation necrosis (RN) are similar to those of local recurrence (LR) of brain metastases (BM). ^11^C–methionine positron emission tomography (MET-PET) is reportedly useful for making a differential diagnosis between LR and RN. In this prospective study, we aimed to investigate the diagnostic performance of MET-PET and the long-term results of subsequent patient management.

**Methods:**

The eligible subjects had enlarging contrast-enhanced lesions (>1 cm) on MR imaging after any form of radiotherapy for BM, suggesting LR or RN. However, it was difficult to differentiate LR from RN in these cases. From August 2013 to February 2017, MET-PET was performed for 37 lesions in 32 eligible patients. Tracer accumulation in the regions of interest was analysed as the standardised uptake value (SUV) and maximal lesion SUV/maximal normal tissue SUV ratios (LNR) were calculated. The cut-off value for LNR was provisionally set at 1.40. Salvage treatment strategies determined based on MET-PET diagnosis and treatment results were investigated. The diagnostic accuracy of MET-PET was evaluated by receiver operating characteristic (ROC) curve analysis.

**Results:**

The median interval from primary radiotherapy to MET-PET was 19 months and radiotherapy had been performed twice or more for 13 lesions. The MET-PET diagnoses were LR in 19 and RN in 18 lesions. The mean values and standard deviation of LNRs for each diagnostic category were 1.70 ± 0.30 and 1.09 ± 0.25, respectively. At the median follow-up time of 18 months, final diagnoses were confirmed histologically for 17 lesions and clinically for 20 lesions. ROC curve analysis indicated the optimal LNR cut-off value to be 1.40 (area under the curve: 0.84), and the sensitivity and specificity were 0.82 and 0.75, respectively. The median survival times of patient groups with LR and RN based on MET-PET diagnosis were 14.8 months and 35.1 months, respectively (*P* = 0.035, log-rank test).

**Conclusions:**

MET-PET showed apparently reliable diagnostic performance for distinguishing between LR and RN. The provisional LNR cut-off value of 1.4 in our institution was found to be appropriate. Limitations of diagnostic accuracy should be recognised in cases with LNR close to this cut-off value.

## Background

The management of patients with brain metastases (BM) has recently become more important because of the increased incidence of these tumors and the prolonged patient survival times that have accompanied improved control of systemic cancers [1–3]. Gadolinium (Gd)-enhanced magnetic resonance (MR) imaging has become a preferred imaging modality not only for early detection of BM but also for evaluation of the efficacy of radiotherapy for BM. Local changes in the area of irradiation application at follow-up, however, are not uncommonly seen on Gd-enhanced and T2-weighted MR imaging [4, 5]. The interpretation of such changes is often difficult and it may even be impossible to differentiate radiation-induced changes from local tumor recurrence [6], which poses a critical dilemma in decision-making for subsequent treatment.

Amino acid tracers such as ^11^C- methionine (MET) are reportedly useful for positron emission tomography (PET), particularly in the field of neuro-oncology, because of high amino acid uptake by tumor tissue with low uptake by normal brain tissue, resulting in an enhanced tumor-to-background contrast [7, 8]. MET-PET studies in primary brain tumors, especially gliomas, have provided promising results, leading to an increase in investigations in the twenty-first century [9, 10]. In contrast, there are few reported evaluations of MET-PET for the imaging of BM [11–14]. Most previous studies investigated imaging changes within already treated BM by focusing on assessment of the diagnostic accuracy of the imaging modalities using receiver-operating characteristic (ROC) curve analysis [11, 12, 14].

The present study aimed to document our early experience with clinical use of MET-PET for distinguishing radiation-induced changes from local tumor recurrence, and to describe in detail the long-term clinical results of modern salvage management based on MET-PET diagnosis. Thus, the diagnostic value and clinical utility of MET-PET imaging for managing patients with BM were critically appraised.

## Methods

### Patient eligibility

The present study was conducted in compliance with the Declaration of Helsinki (sixth revision, 2008), and fulfilled all of the requirements for patient anonymity. The Aizawa Hospital Institutional Review Board (IRB) approved this single center prospective clinical study in July 2013 (No. 2013–049). Written permission was obtained prior to MET-PET from all patients and/or their relatives, allowing the use of personal data for clinical research. Patient records and information were anonymised and de-identified prior to analysis.

The study candidates were limited to patients with BM. Malignant gliomas were excluded from the present study due to the possibility of there being a difference in optimal cut-off values between BM and malignant gliomas [12, 15]. As the routine imaging protocol in our institution, 3-dimensional volumetric gadolinium-enhanced T1-weighted MR images and T2-weighted MR images were obtained for both radiotherapeutic intervention and follow-up imaging studies. In the course of follow-up for BM treated with any type of radiotherapy, including conventional fractionated radiotherapy, stereotactic radiosurgery and particle therapy, lesions with continuous enlargement of Gd-enhanced areas documented on serial MR scans and suspected to be local recurrence (LR) or radiation necrosis (RN), which are difficult to differentiate from each other, were studied using MET-PET. The maximal diameter of a Gd-enhanced area had to be at least 10 mm in order to exclude the possibility of false negative diagnostic errors due to the relatively low spatial resolution of MET-PET. The lesions in which neither LR nor RN could be definitively diagnosed because of insufficient follow-up data were excluded from the present study.

### MET-PET imaging

MET-PET was performed with a Discovery PET/CT 600 scanner (GE Healthcare, Milwaukee, USA) with a spatial resolution of 5.1 mm full width at half maximum. After intravenous injection of about 370 MBq of 11C–methionine, patients were placed in the scanner to assure that slices parallel to the orbitomeatal line could be obtained. After a transmission CT scan had been obtained, a 10-min static emission scan was begun 20 min after the injection. PET images were reconstructed by CT attenuation correction and a 3D ordered subset expected maximisation algorithm (iteration 3, subset 16, field of view 25.6 cm, matrix size of 128 × 128 and slice thickness 3.27 mm).

### MET-PET interpretation

The region of interest (ROI) for lesions was manually located over the area corresponding to the Gd-enhanced area on the MR images. As a normal control, a circular ROI with a diameter of 10 mm was located within the gray matter of the corresponding contralateral side. The quantitative analysis was performed as follows. The maximum standardised uptake values (SUV_max_) within the suspected lesion and within the normal control were measured. The lesion/normal ratios (LNR) were calculated by dividing the SUV_max_ of the lesion by the SUV_max_ of the normal control in order to give priority to detection of a subtle LR mixed with RN. All scans were assessed by an experienced, board-certified, nuclear medicine physician (KO), not involved in any of the treatments for systemic cancer and BM. The cut-off value of the LNR for diagnosis was provisionally set for 1.4, in accordance with previous studies [11, 12, 14]. A LNR exceeding 1.4 was considered to represent LR, a value below 1.4 to mean that the lesion was RN.

### Subsequent management and follow-up

**Fig. 1:**
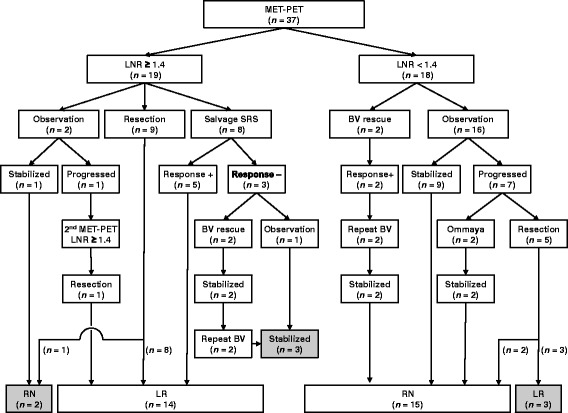
Outcome tree diagram of 37 lesions for which MET-PET was performed to differentiate between LR and RN. Figures in parentheses indicate number of lesions. Halftones indicate the lesions for which MET-PET diagnoses were incorrect or inconclusive

According to the MET-PET diagnosis, subsequent management was determined by a multidisciplinary team in consideration of other clinical factors such as the patient’s age and performance status as well as the anatomical location of the lesion of interest (surgically accessible or not). Details of subsequent management and results were recorded (Fig. 1).

Final clinical diagnoses were determined from surgical specimens, sequential neuroimaging changes and the long-term clinical course secondary to salvage treatment. Shrinkage of the lesion confirmed radiologically after salvage radiotherapy was regarded as LR. A lesion that either remained stable or showed spontaneous shrinkage with no additional treatment on MR imaging follow-up was assumed to be RN. A lesion in which the MET-PET diagnosis could not be confirmed even after adequate follow-up data had been obtained was regarded as a diagnostic failure given the study aim of critical appraisal of MET-PET.

Before closing the research database for analysis in April 2017, the authors updated the follow-up data of patients who had not visited our outpatient department for more than three months. Inquiries about the latest clinical and neuroimaging results and the date and mode of death were made by directly corresponding with the referring physicians and/or the families of deceased patients, with written permission obtained at the time of undertaking MET-PET.

### Statistical analysis

Patient characteristics were compared using Fisher’s exact test for categorical variables and the Mann–Whitney U test for quantitative variables. Receiver operating characteristic (ROC) curve analysis was performed to evaluate the diagnostic capability of MET-PET for differentiating between LR and RN and to determine the optimal cut-off value in our institution, with the weights of false negative and positive classifications being equivalent. The overall survival rates were calculated by the Kaplan-Meier product limit method, based on the interval from the date of MET-PET until the event date. The overall survival of each patient group according to MET-PET diagnosis was compared by log-rank test, wherein a patient with both LR and RN was assigned to the LR group. Proportional hazards regression analysis was not performed in the present study because a too-small ratio of events per variable can lead to inaccurate regression estimates [16].

The statistical processing software package “R” version 3.0.1 (The R Foundation for Statistical Computing, Vienna, Austria) was used for all statistical analyses. A *P*-value <0.05 was considered to indicate a statistically significant difference.

## Results

From August 2013 to February 2017, 33 patients with 38 BM were prospectively registered in the present study. One patient who died of aspiration pneumonia soon after MET-PET was excluded from the analysis. Patient characteristics are presented in detail in Table 1. Of the 32 eligible patients, 19 were male and 13 were female. The median age was 65 years (range: 14–87 years). The primary cancers were of the lung in 22 patients, the breast in 5, the digestive tract in 3, and one each had ethmoid sinus carcinoma and soft tissue sarcoma. The median Karnofsky performance status score at the time of MET-PET was 90 (range: 50–100). All but one patient had received a diagnosis of BM at one of the referring regional hospitals. Twenty-seven patients (84%) had undergone radiotherapeutic intervention using SRS at our institution and the remaining five (16%) had been treated at other institutions and referred to us for MET-PET diagnosis. Fifteen lesions in 13 patients (41%) had received multiple radiotherapeutic interventions prior to MET-PET. The median interval between primary radiotherapy and MET-PET was 18.8 months (range: 4–120 months). Fifteen patients (47%) had been receiving systemic chemotherapy at the time of MET-PET planning, but none had been administered bevacizumab (BV), a monoclonal antibody against vascular endothelial growth factor.

**Table 1:** Baseline demographic and clinical characteristics

Characteristic	Value
Sex (male/female)	19/13
Age^a^ (years), median (range)	65 (14–87)
Primary cancer	
Non-small cell lung cancer	
EGFR wild-type	14
EGFR mutant	5
Small cell lung cancer	3
Breast cancer	
HER2-positive	4
HER2-negative	1
Gastrointestinal cancer	2
Oesophageal cancer	1
Sinonasal adenoid cystic carcinoma	1
Rhabdomyosarcoma	1
KPS^a^, median (range)	90 (50–100)
Neurological deficits^a^	24 (75%)
RTOG-RPA Class^a^ (I/II/III)	8/18/6
Multiple BM^a^	16 (50%)
Prior radiotherapy (per lesion)	
Proton therapy	1
SRS	21
WBRT + SRS	3
SRS × 2	9
SRS × 3	2
SRS × 4	1
Time from primary radiotherapy to MET-PET (months), median (range)	18.8 (4–120)

EGFR epidermal growth factor receptor, HER human epidermal growth factor, KPS Karnofsky performance status, RTOG radiation treatment oncology group, RPA recursive partitioning analysis, BM brain metastases, ^a^updated status at the time of MET-PET, SRS stereotactic radiosurgery, WBRT whole brain radiotherapy, MET-PET 11C–methionine positron emission tomography

### MET-PET diagnosis and salvage management

Nineteen tumors were diagnosed as LR and 18 as RN by MET-PET. The mean value and standard deviation of LNRs for each diagnostic category were 1.70 ± 0.30 and 1.09 ± 0.25, respectively. Comparison of baseline characteristics between the two groups revealed neurological symptoms caused by the lesion of interest to be significantly more frequent in the LR than in the RN group (P = 0.011), while the time from primary radiotherapy to MET-PET was significantly longer in the RN group (median: 24.9 months) than in the LR group (median: 14.3 months) (*P* = 0.046) (Table 2).

**Table 2:** Difference in clinical characteristics between MET-PET diagnosis groups

Characteristic	LNR ≥ 1.4 (*n* = 19)	LNR < 1.4 (*n* = 13)	*P* value
Sex (male/female)	12/7	7/6	0.72
Age^a^ (years), median (range)	67 (14–87)	63 (49–79)	0.45
KPS^a^, median (range)	80 (50–100)	90 (60–100)	0.26
Neurological deficit^a^	15 (79%)	9 (69%)	0.011
RTOG-RPA Class^a^ (I/II/III)	4/11/4	4/7/2	0.79
Multiple BM^a^	11 (58%)	5 (38%)	0.47
Repeat prior radiotherapy	7 (37%)	6 (46%)	0.72
Time from primary radiotherapy to MET-PET (months), median (range)	14.3 (4–120)	24.9 (6–111)	0.046

MET-PET ^11^C–methionine positron emission tomography, LNR lesion/normal ratios, KPS Karnofsky performance status, RTOG radiation treatment oncology group, RPA recursive partitioning analysis, BM brain metastases, ^a^updated status at the time of MET-PET

**Fig. 2:**
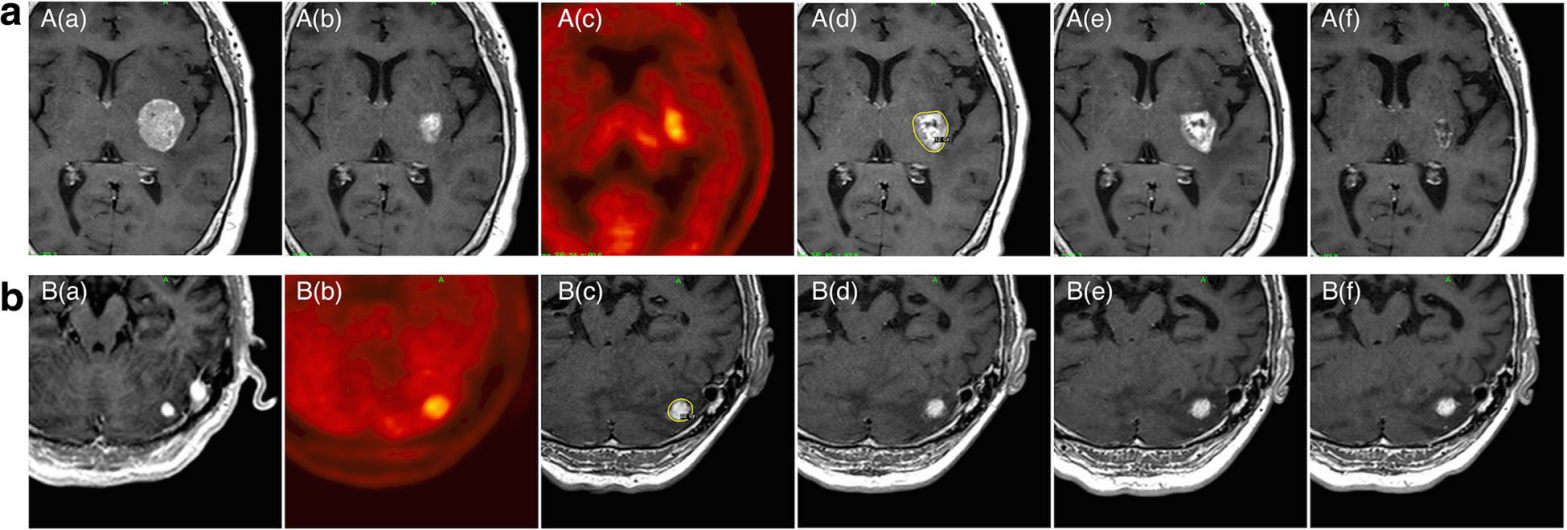
Serial axial Gd-enhanced MR images and MET-PET images of two cases in which MET-PET diagnoses could not be confirmed even with sufficient follow-up after salvage treatment. **a**: 60s–year-old-woman with multiple brain metastases from breast cancer. (a) Gd-enhanced MR image obtained at the time of the initial SRS. (b) Gd-enhanced MR image obtained three months after SRS. (c) MET-PET image obtained 8 months after initial SRS. The LNR was 1.67. (d) Gd-enhanced MR image obtained at the time of the second SRS. The yellow line represents the prescription isodose volumes (12 Gy at 50%). (e) Gd-enhanced MR image obtained 1 month after the second SRS before BV rescue. Re-irradiation caused neurological worsening and perifocal oedema. (f) Latest follow-up Gd-enhanced MR image obtained 20 months after MET-PET. Repeat BV therapy resulted in symptomatic relief and radiological stabilisation. **b**: 70s–year-old-man with solitary cerebellar metastasis from gastric cancer. (a) Gd-enhanced MR image obtained at the time of the initial SRS. (b) MET-PET image obtained 19 months after the initial SRS. The LNR was 1.50. (c) Gd-enhanced MR image obtained at the time of the second SRS. The yellow line represents the prescription isodose volumes (22 Gy at 50%). (d) Gd-enhanced MR image obtained 12 months after the second SRS. Re-irradiation caused cerebellar ataxia, requiring temporary conservative treatment with oral steroids. (e) Gd-enhanced MR image obtained 26 months after the second SRS. (f) Latest follow-up Gd-enhanced MR image obtained 36 months after MET-PET. Symptomatic relief and radiological stabilisation were maintained during long-term observation

Subsequent management based on MET-PET diagnosis is shown in Fig. 1. Of 19 LR, microsurgical resection and SRS were performed in 9 and 8 lesions, respectively. True tumor recurrence with various degrees of necrotic tissue was histologically confirmed in 8 of 9 surgical specimens. The rest of the lesion, previously irradiated twice, was microscopically diagnosed as pure RN. After salvage SRS, three of eight lesions showed no evident decrease in Gd-enhanced areas or perifocal oedema. Two of these patients, one with HER-2 positive breast cancer and other with EGFR wild-type lung adenocarcinoma, experienced neurological worsening and needed salvage therapy using repeat BV, resulting in immediate and durable symptomatic relief and radiological stabilisation for more than 20 months (Fig. 2a). The other patients were cautiously observed with temporary use of oral steroids, showing Gd-enhanced areas and perifocal oedema which remained stable for more than 3 years (Fig. 2b). We strategically chose an observation policy in two cases diagnosed as having LR on MET-PET. One eventually showed disease progression and was confirmed to have a true recurrence after surgical intervention, and the other exhibited a self-limiting course after MET-PET, which was consistent with RN. Of the 18 with RN, two patients with moderate to severe neurological complications required repeat BV therapy, which produced rapid and substantial symptom relief without relapse. Some of the remaining 16 patients were initially observed or managed conservatively with low-dose oral steroids. Seven of these cases, however, showed gradual progression and ultimately needed salvage surgical treatment, and 3 of these lesions were histologically confirmed to be LR.

### ROC curve analysis

**Fig. 3:**
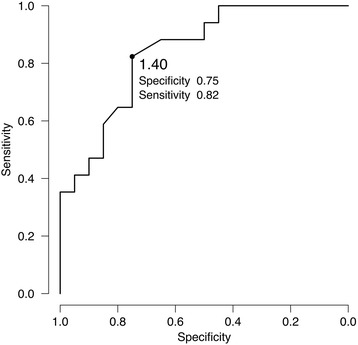
ROC curve for LNR for MET-PET diagnosis. The LNR cut-off value of 1.40 (dot) provided the best specificity and sensitivity for differentiating between LR and LN, 0.75 and 0.82, respectively. The highest area under the ROC curve was 0.84 (95% CI: 0.71–0.97)

The ROC curve analysis for LNR is shown in Fig. 3. The cut-off value for LNR of 1.40 provided the optimal sensitivity and specificity for differentiating LR from RN, 0.82 and 0.75, respectively. The highest area under the ROC curve was 0.84 (95% CI: 0.71–0.97). Seven of the 8 lesions misdiagnosed on MET-PET had LNR values close to the provisional cut-off point (within the range of 1.4 ± 0.2).

**Fig. 4:**
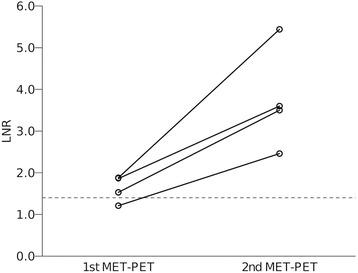
Comparison of LNRs between the first and second MET-PET in 4 patients. The LNR of the second scan (median: 3.55) showed a marked increase as compared to that on the first scan (median: 1.70) (*P* = 0.039, Paired t test)

A second MET-PET was necessary in 4 patients because of uncertainties in their clinical courses even after salvage management based on the diagnosis made using the first MET-PET. The LNR of the second scan (median: 3.55) showed an obvious increase above that of the first scan (median: 1.70) (*P* = 0.039, Paired t test) (Fig. 4) and subsequent treatment confirmed true recurrence in all 4 cases.

### Patient survival

**Fig. 5:**
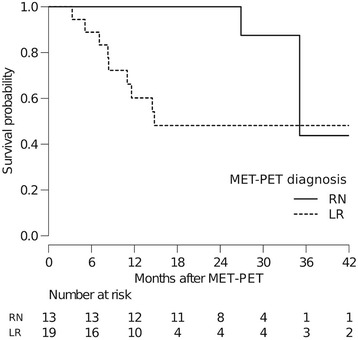
Kaplan –Meier curves showing the survival estimates for patients with LR (dotted line) and RN (solid line) according to MET-PET diagnosis. The median survival times in the LR and RN groups based on MET-PET diagnosis were 14.8 months (95% CI: 8.4 – NR) and 35.1 months (95% CI: 26.9 –NR), respectively (*P* = 0.035, log-rank test)

The median follow-up interval of all patients after MET-PET was 17.5 months (range: 3–45). At the time of assessment, 20 patients (63%) were alive and 12 (37%) had died. Nine of the 12 deceased patients had died of CNS disease progression. The median survival time was 45.4 months (95% CI: 26.9 – NR (not reached)) (Fig. 5). The median survival times in the LR and RN groups based on MET-PET diagnosis were 14.8 months (95% CI: 8.4 – NR) and 35.1 months (95% CI: 26.9 –NR), respectively (*P* = 0.035, log-rank test) (Fig. 5).

## Discussion

After any type of highly focused radiotherapy for BM, one of the toxicities of greatest concern is delayed RN. Because recent advancements in systemic therapy such as targeted therapy have prolonged the survival of even patients with BM [1–3], the importance of management for delayed neurotoxicity and recurrence is anticipated to further increase. In the follow-up of patients with BM treated with radiotherapy, it is a matter of major importance to differentiate between delayed RN and LR either clinically or with MR imaging [6]. The use of stereotactic biopsy for histological assessment of post-radiotherapeutic changes in MR imaging can be regarded as a viable diagnostic option [13], though it is somewhat invasive and not always feasible. At present, many imaging methods are practiced, including MR imaging (dynamic susceptibility contrast perfusion [17], diffusion [18] and proton MR spectroscopy [19]) and nuclear medicine techniques (18F–fluorodeoxyglucose PET [20] and 201-thallium single-photon emission computerised tomography [21]). The active uptake of amino acids in viable tumor cells is different from that in radiation-induced changes wherein only passive diffusion across the damaged blood-brain barrier occurs [7–10]. Thus, MET-PET is theoretically expected to reveal metabolic information in addition to morphological changes, resulting in high BM detection rates and clear lesion delineation [22]. Our early experience with MET-PET appeared to yield reliable and reproducible diagnostic performance for differentiating between LR and RN, when compared to areas under the ROC curves of previous studies by other investigators, although there were some differences in the calculation methods used between studies (Table 3). The provisional LNR cut-off value of 1.4 in our institution was herein confirmed to be appropriate. Thus, we will not change our diagnostic criteria.

**Table 3:** Comparison of qualitative tests of MET-PET for differentiation between LR and RN in BM

First author & year	No. of lesions	LNR cut-off value	Sensitivity (%)	Specificity (%)	AUC
Tsuyuguchi, 2005	21	1.42^a^	78	100	NR
Terakawa, 2008	51	1.41^a^	79	75	0.78
Minamimoto, 2015	42	1.30^b^	82	86	0.89
Present study, 2017	37	1.40^b^	82	75	0.84

MET-PET ^11^C–methionine positron emission tomography, LR local recurrence, RN radiation necrosis, BM brain metastases, LNR lesion/normal ratios, AUC area under the curve, NR not reported

^a^SUV_mean_ (lesion)/SUV_mean_ (reference), ^b^SUV_max_ (lesion)/SUV_max_ (reference)

Several previous retrospective studies of MET-PET for BM focused on its diagnostic accuracy and provided an optimal cut-off value for diagnosis but, unfortunately, did not provide subsequent management details. Such specific details are often of critical importance to physicians caring for BM patients. The authors sought to provide information useful for physicians on how to manage such refractory situations, by investigating not only the diagnostic accuracy of MET-PET but also the long-term results of salvage management. We believe this novel viewpoint to be the core value of the present work.

This is the first report to demonstrate that MET-PET can predict the patient’s survival as well as providing the immediate diagnosis. This exploratory insight has, in our opinion, clinical significance and can be regarded as relevant because RN follows a self-limited course in most cases while, in contrast, LR can lead to neurological death. In fact, we observed that 9 patients diagnosed with LR on MET-PET ultimately succumbed to central nervous system disease progression despite multimodal treatments. The overall survival time after MET-PET was also found to be prolonged in RN as compared to LR cases. We speculate that this might, at least in part, be attributable to the patients receiving MET-PET having been self-selected to do well by virtue of having had time to develop neuroimaging changes and not dying of their systemic disease. This potential patient population bias should be noted and caution must be exercised in the generalisation of our present findings.

There are limitations in the accuracy of imaging diagnosis. LNR values close to 1.4 should be regarded as a “grey zone” with relatively low reliability and the possibility of the initial diagnosis ultimately being found to be incorrect. As demonstrated herein, some of the lesions diagnosed as RN by MET-PET were eventually confirmed to be LR, based on meticulous observation [23]. The second MET-PET was shown to be a meaningful option for detecting longitudinal metabolic progression, but given that the LNR values were significantly higher on the second MET-PET, a lesion in such a case might simply be regarded as LR. A delay in accurate diagnosis can, in turn, lead to delayed initiation of salvage management. Given that the process of RN might, in part, represent a progressive destructive cascade, the timing of MET-PET planning is of particular importance for effective salvage management aimed at preventing further histologic injury. In addition to MET-PET imaging findings, we should also focus on patient’s progressively worsening neurological symptoms and the shorter time interval from radiotherapy to MET-PET as potentially useful references for the diagnosis of LR, as shown in Table 2.

Even with salvage management and long-term follow-up, the validity of MET-PET diagnosis could not be determined in 3 patients (Fig. 2). We speculate that the limitations of not only MET-PET but also other neuroimaging techniques for differentiation between LR and RN might be attributable to there being three rather than two possible diagnoses; LR, RN and pathology combining these two. A series of microscopic analyses of recurrent BM after SRS demonstrated that as many as 10 to 78% of lesions were diagnosed as showing mixed pathology with various degrees of viable tumor and necrotic tissue [6, 24–27]. The management of both LR and RN poses a significant therapeutic dilemma, if surgical resection is not feasible, and effective therapies have yet to be established. It is noteworthy that modern combined management using SRS followed by adjuvant BV might be a viable and durable treatment option, even for such complex conditions as in some of the patients reported herein. Although evidence supporting the remarkable effects of BV for refractory cerebral RN has recently been accumulating [28–31], BV treatment is not yet recognised as a standard of care for RN and is not currently reimbursed by our public healthcare system, necessitating that the treatment indications be strictly limited to cases having no alternative but to undergo such an unproven treatment given its potential toxicity. Optimisation of multimodal treatment using antiangiogenic drugs merits further research.

The results of our present study must be interpreted with caution. The subjects were a heterogeneous group. The number of subjects was small and the lack of adequate statistical power may have resulted in the dataset being underpowered to appropriately assess hypotheses and potential prognostic factors. It should also be noted that it remains difficult to make an accurate diagnosis in some cases with the clinical and radiographic methods employed herein. Thus, we plan to accumulate further experience with this imaging modality, in hopes of establishing more efficient diagnostic and salvage treatment regimens.

## Conclusions

MET-PET appeared to have a reliable diagnostic capability for distinguishing between LR and RN after radiotherapy for BM. The provisional LNR cut-off value of 1.4 in our institution was found to be appropriate. Diagnostic accuracy limitations should be recognised in cases with LNR close to this cut-off value. An exploratory analysis raised the possibility of MET-PET diagnosis predicting patient survival.

## Abbreviations

AUC: Area under the curve
BM: Brain metastases
CI: Confidence interval
EGFR: Epidermal growth factor receptor
Gd: Gadolinium
HER: Human epidermal growth factor
KPS: Karnofsky performance status
LINAC: Linear accelerator
LNR: Lesion/normal ratios
LR: Local recurrence
MET-PET: ^11^C–methionine positron emission tomography
MR: Magnetic resonance
RN: Radiation necrosis
ROC: Receiver operating characteristic
RPA: Recursive partitioning analysis
RTOG: Radiation treatment oncology group
SRS: Stereotactic radiosurgery
SUV: Standardised uptake value
WBRT: Whole brain radiotherapy
